# Trans-Inclusive Communication and Self-Perceived Barriers to It, as Reported by Doctors—A Mixed-Methods Survey in Germany

**DOI:** 10.3390/healthcare12070707

**Published:** 2024-03-23

**Authors:** Rieka von der Warth, Mirjam Körner, Erik Farin-Glattacker

**Affiliations:** 1 Section of Health Care Research and Rehabilitation Research, Institute of Medical Biometry and Statistics, Medical Center—University of Freiburg, Faculty of Medicine, University of Freiburg, 79106 Freiburg, Germany; erik.farin@uniklinik-freiburg.de; 2 Institute of Medical Psychology and Medical Sociology, University of Freiburg, 79104 Freiburg, Germany; mirjam.koerner@mail.mps-uni-freiburg.de

**Keywords:** trans-inclusive, transgender, gender diverse, health communication, doctors’ behaviour, patient-centred care

## Abstract

The majority of transgender and gender-nonconforming people (TGNC) report negative experiences with doctors in the healthcare system. As there is little knowledge about the communication behaviour of doctors towards TGNC, this survey aimed to assess the self-reported trans-inclusive communication of doctors and their willingness to communicate trans-inclusively, as well as their self-perceived barriers to it. A mixed-methods survey was applied for this. Firstly, we measured self-reported trans-inclusive communication behaviour based on the CommTrans questionnaire. Based on this, the overall willingness, as well as self-perceived barriers (qualitative) to communication, were assessed. In total, N = 57 doctors took part in the survey. Most participants reported not introducing themselves using pronouns (79.4%). Of these, 61.4% said that they would not be able to do this in the future either. Perceived barriers were classified into the following eight categories: necessity, sample-dependency, habit, structural barriers in practice, uncertainties in dealing with the topic, limits of patient-centredness, gender as a binary concept, and transphobia. In summary, doctors in Germany show different degrees of trans-inclusive communication. It is likely that this has a negative effect on TGNC, their health and access to the healthcare system.

## 1. Introduction

The umbrella term transgender and gender-nonconforming people (TGNC) is used for individuals who do not identify fully and constantly with the sex they have been assigned at birth [1]. TGNC individuals might identify with a different gender recognised by society (male/female) [2], or they might associate themselves with both, between or neither male nor female [2]. Studies suggest that about 80% of all TGNC individuals identify with a binary gender concept (male/female), whereas 20% identify as gender-diverse, such as, e.g., two-spirit, genderfluid, and agender [3].

TGNC individuals encounter frequent stigma at different levels of the healthcare system, with one being the interpersonal level between healthcare staff and patients [4]. One important factor associated with this is that doctors and other medical staff are not able to deal with the ambivalence between appearances, personal details, and bodies because a binary cis-gender system in the medical field is assumed [5,6]. Cis-gendered are individuals who identify with the gender they were assigned at birth. Furthermore, patients report that doctors have stereotypical assumptions about TGNC individuals and make care decisions based on this, for example, with regard to sexual health [7]. In this context, the so-called „Trans-Broken Arm Syndrome” further describes the phenomenon that doctors misattribute acute medical complaints to the patient’s gender identity or medical transition or might ask invasive and unnecessary questions about the patient’s gender identity [8]. Overall, many TGNC individuals report negative experiences with staff in the health system [9], including physical violence [10]. In turn, a TGNC individual might develop a fear of doctors [11], resulting in an avoidance of healthcare services [12,13]. Additionally, experienced stigma by doctors is related to depression and anxiety in TGNC people [14,15].

At the same time, little is known about the doctors’ knowledge of TGNC individuals, with TGNC individuals themselves identifying knowledge gaps among doctors in medical care [16]. With regard to care for queer people, which includes TGNC individuals, a literature review found a lack of competence and the training of doctors to provide care [17]. The lack of competence increases further when caring for TGNC people is investigated individually [17]. With regard to doctors’ attitudes, a distinction must be made between general gender-related beliefs, interpersonal comfort in contact with TGNC, and the attribution of human rights [18,19]. These dimensions overlap with the concept of transphobia [20], which plays an important role in the provision of healthcare, alongside formal knowledge about TGNC [21]. Due to transphobic beliefs, some doctors even refuse to provide care [16,22].

Simultaneously, TGNC individuals have numerous specific communication preferences, both on a gender-specific level and a general interaction level [23]. Patient communication preferences are usually described as the communication style patients wish doctors to show during medical consultations [24]. Fulfilled patient communication preferences are known to be associated with a good patient–doctor relationship [25], shared decision making, and patient engagement [26]. Previous research suggests that patient communication preferences are particularly important for minority populations [27] and that supportive interactions with doctors actually result in better mental health in TGNC individuals [28].

However, little is known about the specific trans-inclusive communication behaviours of doctors, their willingness to show such behaviour, as well as their self-perceived barriers to trans-inclusive communication. Yet, marginalised patient groups, such as TGNC individuals, may have a different concept of cultural health communication, and knowledge of the reciprocal nature of communication is critical for eliminating health disparities [29,30]. We, therefore, conducted a mixed-method survey to assess all of these named aspects. We used the quantitative part of the survey to assess self-reported communication behaviour and willingness to communicate trans-inclusively. The qualitative survey part assessed the corresponding barriers to this. Furthermore, we assessed whether there are any group differences between those doctors who are willing to show trans-inclusive communication behaviour and those who are not, based on the following groups: sex, age, finished residency, years of clinical experience, and level of transphobia.

## 2. Materials and Methods

Participant recruitment was conducted at the Medical Centre—University of Freiburg. The ethical approval was granted by the Ethics Council of the University of Freiburg (Approval Number: 21-1609). The study was registered in the German Clinical Trial Register (DRKS00026249).

### 2.1. Recruitment

The online survey was carried out between April and August 2023 via the RedCap platform (Version 14.0.15) [31,32]. Recruitment took place using a snowball system. An invitation to participate was published on the intranet of the Medical Centre—University of Freiburg—and on its social media platforms. Furthermore, various medical professional associations (e.g., the German Association for Social Medicine and the German Association for Medical Sociology) were contacted with the request to publish the study call. For this purpose, a social media tile was created, which summarised all the information for interested individuals. These individuals then had the option to follow an internet link to access the survey. Before those interested were referred to the survey, they were informed about it in writing and explicit consent was obtained. Detailed study information was available to download. After consent, the inclusion criteria were queried, which were a legal age of at least 18 years, a completed medical license, and current clinical activity. The survey terminated if participants did not agree to the study or did not meet the inclusion criteria.

### 2.2. Sample

In total, 214 interested individuals started the survey, of whom n = 89 agreed to participate. Questions regarding their trans-inclusive communication were answered by up to N = 69 doctors, while n = 59 doctors finished the survey. However, two cases had to be removed due to ambiguous data, resulting in a final sample of n = 57. Sociodemographic data were assessed with inter alia sex and gender, years of clinical experience, and medical specialisation.

Participants were, on average, 36.6 years old (Std. Deviation = 8.6 years). All the participants identified with the sex they were assigned at birth, with 65% being female. At 51.8%, slightly more than half of the participants had already completed at least one residency training, with the majority of participants in general medicine, internal medicine, and surgery. In total, 86% of all the participants stated that they already had at least one instance of contact with a person who identified as TGNC. More information on participants can be found in Table 1.

### 2.3. Instruments and Study Design

Trans-inclusive communication behaviour was assessed using a reformulated version of the CommTrans questionnaire [33]. The CommTrans questionnaire is a new questionnaire assessing the communication preferences of TGNC individuals during medical consultations. The development of the CommTrans questionnaire was based on a qualitative interview study with TGNC [23]. The final questionnaire comprises two scales with nine items in total: “emotional resonance” and “gender-specific communication” [33]. The questionnaire was developed for research and practice and aims to facilitate doctors’ behaviour and processes in the healthcare system.

In addition to the items in the original CommTrans questionnaire, all the items, which were excluded during its development due to ceiling effects (high preference) were included in this survey study. This was based on the consideration that these items had particular subjective importance to TGNC individuals in medical care. Items in this survey can be divided into general interaction communication (e.g., “I am generally happy for my patients when their treatment progresses positively”) and gender-specific communication (“I always ask my patients for their pronouns.”). All the items were reformulated in such a way that they corresponded to a behaviour-orientated statement. Answers were given on a Likert scale from 0/strongly disagree to 6/strongly agree. Based on the items’ answers, a prompting command was programmed into the survey. Participants who stated they did not show the behaviour in question in their clinical practice (response categories 0 and 1) were then asked the same question again. However, when asking for the second time, the query was: “Will you generally be able to communicate this way in the future?”. The answer option here was dichotomous with yes/no.

**Figure 1:**
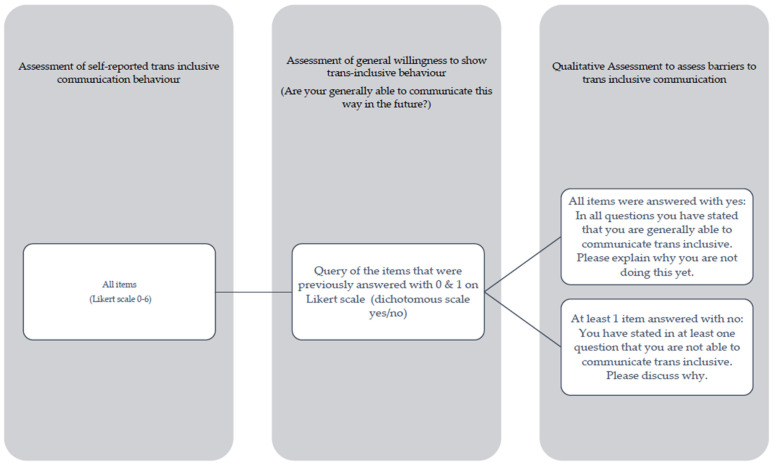
Process of the mixed-methods study using prompting technique.

This answer, in turn, had an impact on the qualitative part of the mixed-methods survey, with the aim of assessing self-perceived barriers to trans-inclusive communication. If the participants answered “yes” to all the items, they were asked why they did not yet communicate trans-inclusively. If at least one item was answered with “no,” the participants were asked to discuss why they would also not engage in trans-inclusive communication in the future. The process of the entire mixed-method survey is shown in detail in Figure 1.

Furthermore, we included the transphobia scale by Nagoshi, Adams [20]. For the purpose of this survey, we translated the screener into German using a forward–backwards translation method [34]. Two native German speakers who used to live and work in an English-speaking country led the forward translation. Two native English speakers, who both spoke fluent German, in turn, led the backward translation method. Differences in translation were resolved in the discussion.

Answers to the nine items (e.g., “When I meet someone, it is important for me to be able to identify them as a man or a woman.” or “I believe that a person can never change their gender.”) are given on a 7-point Likert scale from 1/“I don´t agree at all” to 7/“I agree totally”. For analysis, we created a mean score, which ranged between 1 and 7. Cronbach´s Alpha was 0.73 in our survey.

### 2.4. Analysis

The results of the quantitative items are shown descriptively. In this way, specific trans-inclusive communication can be shown independently and discussed. Furthermore, we investigated group differences in participants, who stated they would generally be able to communicate trans inclusively in the future, compared to those who did not—we used Chi-square tests and *t*-tests. Group variables were sex, age, finished residency, years of clinical experience, and level of transphobia.

Qualitative answers from the last part of the survey were grouped into different categories. The grouping of free-text answers was indicative based on the answers given. Both of the qualitative questions asked were analysed separately but were later compared to each other to detect differences between the two groups of doctors. Example answers for each category are displayed, which were translated from German to English by the authors of this paper.

## 3. Results

### 3.1. Self-Reported Trans-Inclusive Communication

As regards the items on general interaction communication, the majority of participating doctors stated they already communicated trans-inclusively. Three participants disagreed with the statement that “I generally enable my patients to ask all their questions”, which was the highest rate of non-agreement in this block. Of these, two participants stated they would not be able to perform this in the future either.

A different picture emerged with regard to gender-specific communication. Participants were least likely to introduce themselves using their pronouns (79.4%). Of these, 61.4% said that they would not be willing/able to do so in the future. In comparison, 69.4% (n = 43) of the participants stated that they would not avoid gender-specific language in the initial consultation, but n = 20 doctors generally felt they were able to do so. An overview of all the items and participants’ answers is given in Table 2.

### 3.2. Self-Reported Barriers to Trans-Inclusive Communication

In total, 23 participants said that they were able to comply with all the queried trans-inclusive communication and were, therefore, asked why they were currently not doing so. At the same time, 32 participants answered with “no—I am not/will not be able to do this in the future” for at least one question. Participants in these two groups did not differ in sex, age, finished residency, and years of clinical experience. However, participants in the group who were not able to show trans-inclusive communication behaviour had a significantly higher score on the transphobia scale (mean = 2.47, SE = 1.01) compared to those who felt able to show all the requested behaviour (mean = 1.71, SE = 0.55; *t*(48.06) = 3.54, *p* < 0.001).

Of the 23 doctors stating they saw themselves as capable of trans-inclusive communication, 19 doctors took part in the qualitative part of the survey. Of the 32 doctors stating they could not show the requested communication behaviour in at least one case, 23 participants answered the qualitative survey item. Overall, answers in both groups were similar, with the following six overlapping categories: necessity, sample-dependency, habit, structural barriers in practice, uncertainties in dealing with the topic, and limits of patient-centredness.

The most commonly named barrier as to why doctors did not show the requested behaviour was that they felt that there was no need. The perceived low necessity was mainly justified by the fact that there was little contact with TGNC individuals and that the prevalence of the TGNC group was low. Others stated that mentioning one’s own pronouns, in particular, was not relevant if one was to introduce oneself with a gendered job title. Some doctors stated that all the relevant information, such as pronouns and gender, is already included in the referral letters from other doctors, and, thus, there is no need to ask for them. The two topics of sample-dependence and habit were closely linked to the topic of necessity. In the topic of sample-dependency, for example, it was mentioned that they mainly treat older patients who would not understand the questions about gender and pronouns and, if in doubt, might even react to it with anger.

It was further mentioned that there would be structural barriers in one’s own practice. For example, because one had no influence on the website and processes and/or because the management level had no understanding of the topic. Another important topic was that one did not know how to best implement certain trans-inclusive communication behaviours without appearing disrespectful. In relation to gender-specific language, the answers showed that the requested communication behaviour was previously partly unknown to the participants. However, some participants stated that they would be happy to show it now that they knew of it.

A few answers were also given specifically to general interaction behaviours, and these referred to the fact that patient-centredness had its limits because doctors had more knowledge about interventions, and treatments had to be evidence-based.

Two further categories emerged in the group of doctors who said they would not be able to communicate trans inclusively in the future: gender as a binary concept and transphobia. In the first category, doctors stated they would assume a binary gender, as this was the biological given possibility, and they would use this as the base for their communication behaviour. Additionally, two doctors gave answers which are categorised as transphobic by saying TGNC people have a mental illness, for example.

An overview of all categories and example answers is given in Table 3.

## 4. Discussion

In this survey, we assessed the trans-inclusive communication behaviours of doctors towards TGNC as well as their self-perceived barriers to it. For this purpose, we conducted a mixed-method survey. We thereby used the quantitative part of the survey to assess self-reported behaviour concerning trans-inclusive communication preferences. The qualitative survey part was used to assess the corresponding barriers. We found that doctors differed in their assessment of general interaction communications compared to gender-specific communication.

As far as general interaction communication is concerned, most participants stated that they already showed the behaviour in question in their daily work. Only a few participants in four different communication behaviours said that they would not behave as is preferred by TGNC individuals. This is in line with the literature, with doctors valuing patient-centred communication highly [35] and feeling like they already communicate in a patient-centred way, thus enhancing shared decision making in clinical practice [36]. Yet, we found one qualitative category regarding the limits of patient-centred care and communication and the need to recommend evidence-based treatment. Even though we cannot draw conclusions about the actual behaviour of doctors participating in our survey, it is known that the borders between the patient-centred recommendation of evidence-based treatments and paternalistic persuasion are fluid in practice [37]. Thus, doctors might be at risk of reverting to a paternalistic manner in their daily practice while recommending treatments [38]. This could lead to patients undergoing treatment against their preferences, which is of special relevance for gender-affirming care in TGNC individuals, as an individual’s gender experience should be focused upon during transition treatments [39,40].

Concerning gender-specific communication, a high proportion of doctors stated they would not communicate trans-inclusively and did not feel like they could do so in the future. Two important barriers to trans-inclusive communication were perceived as low necessity and habit. In particular, the perceived low necessity might be problematic, as patient-centred communication, which is adjusted to the individual, is known to affect, inter alia, the self-management of patients, inspiring greater trust in doctors and a less frequent change in doctors [25,41]. The latter might be of special relevance for TGNC individuals, who are known to avoid healthcare [12]. In fact, it is already known that good provider-patient interactions with TGNC individuals are associated with better healthcare outcomes [28], thus highlighting the necessity of doctors’ trans-inclusive communication. Doctors in this survey said they perceived a low necessity because they had little contact with TGNC individuals in their daily practice. Previous studies reported similarly high proportions of known contacts in the past; however, most doctors have not had frequent contact within the last few years [42,43]. This is especially difficult, as learning patient-centred communication is more appropriately addressed by contextualised learning [44]. Doctors are known to rate trans-contextualised teaching, which is provided by the peer group, as valuable [45]. This approach might further decrease transphobia and insecurities, as having more contact with and education about TGNC individuals is associated with less prejudiced beliefs [46]. One further reported barrier was the sample-dependency on communication and the complexity of adjusting to it. In fact, being able to adjust to a single patient is a core characteristic of a skilled communicator in healthcare [47] and is necessary to achieve patient-centred outcomes [48,49]. This further highlights the need for contextualised learning about the TGNC group.

Lastly, two main barriers were found only with those doctors who stated that they were not able to show trans-inclusive communication behaviour: gender as a binary concept and transphobia. Those doctors also had a significantly higher score on the transphobia scale, underlining the results of the qualitative survey. These two barriers should be discussed in context with each other. However, we decided to differentiate between these two as openly harmful statements (gender incongruence as mental illness), displaying a different degree of trans-exclusive behaviour, which is likely to negatively affect TGNC health. Transphobia plays an important role in the provision of healthcare alongside formal knowledge about TGNC [21], and doctors might even refuse care [16,22]. After all, even subtle trans-exclusive communication and practices due to a binary gender concept might have a negative effect on TGNC individuals [6,50] and create further barriers in the provision of healthcare [51].

### Strengths and Limitations

In this paper, we provide an insight into the trans-inclusive communication of doctors in Germany. We have been able to show the self-reported communication, willingness, and barriers to preferred behaviour needed by TGNC individuals. However, some limitations should be discussed. Firstly, our sample is relatively small and includes a wide variety of medical specialities. The latter is due to our sampling method, where we addressed all the doctors independent of their training in our study invitation. Thus, the results of this survey cannot be generalised to a specific medical field in Germany. Furthermore, we must assume a sample bias, as we had a relatively young and female sample. Both younger and female doctors have previously been shown to be more open-minded, as well as more competent, with respect to trans care [42,43,52]. At the same time, a recent systematic review showed that most mental health professionals, who are also included in this survey, nowadays hold positive attitudes and beliefs [53].

Furthermore, we assessed transphobia using a self-translated version of the transphobia scale [20]. Even though we found Cronbach´s Alpha to be good at α = 0.73 and we were able to differentiate those doctors who were willing to show trans-inclusive behaviour from those who were not, we must assume a bias in this translated version of the scale. We assume this bias, as the mean score in our survey is not comparable to the score published in the original publication of the scale [20]. Our participants showed a relatively low level of transphobia, with a mean score of 2.16 on a scale from one to seven. However, this low mean score could also be related to the overall sample bias.

## 5. Conclusions

Doctors in Germany seem to show different degrees of trans-inclusive communication, with doctors reporting more trans-inclusive behaviour in general communication aspects compared to gender-specific communication. As a main barrier to trans-inclusive communication, doctors stated that there was a low perceived necessity. An important reason for this might be the lack of contextualised training for doctors. Thus, based on our results, we support the claim for more trans-inclusive medical teaching and the strengthening of trans care. This way, barriers for TGNC individuals to access healthcare might be reduced, which, in turn, can have a positive effect on their health.

## Figures and Tables

**Table 1:** Sociodemographic data of participants.

**Sex (assigned at birth)**		
Female	37	64.9
Male	20	35.1
**Completed residency**		
Yes	29	51.8
No	27	48.2
**Medical speciality**		
General medicine	14	26.9
Anaesthesiology	3	5.8
Surgery	10	19.2
General Surgery	1	
Cardiac Surgery	1	
Oral and maxillofacial surgery	1	
Orthopaedics and trauma surgery	2	
Plastic and aesthetic surgery	3	
Thoracic surgery	1	
Visceral surgery	1	
Gynaecology and obstetrics	1	1.9
Otorhinolaryngology	1	1.9
Human genetics	1	1.9
Internal medicine	10	19.2
Infectiology	1	
Cardiology	2	
Orthodontics	1	1.9
Paediatrics	3	5.8
Neurology	1	1.9
Psychiatry and psychotherapy	3	5.8
Radiology	2	3.9
Urology	1	1.9
Dentistry	1	1.9
**Clinical setting**		
Stationary	26	45.6
Partial stationary	2	3.5
Ambulant	28	49.1
Others	1	1.8
**Known contact with transgender and gender-nonconforming people**		
Yes	49	86.0
No	2	3.5
I don’t know	6	10.5
	Mean	Std. Deviation
Age in years	36.60	8.7
Professional experience since license (in years)	8.61	7.7
Transphobia scale	2.16	1.0

**Table 2:** Doctors self-reported trans-inclusive behaviour as well as their willingness.

		I Already Communicate like This with My Patients.				Are You Generally Able to Communicate This Way in the Future?			
		Disagree (0 and 1)		Agree (2–6)		Yes		No	
		N	%	N	%	N	%	N	%
General interaction communication	I am generally happy for my patients when their treatment progresses positively. *	/	/	63	100				
	I always manage to encourage my patients when they are nervous. *	/	/	64	100				
	I always manage to give my patients the feeling that I am aware of how important the treatment is for them. *	/	/	62	100				
	I always manage to give my patients the feeling that the treatment is individually tailored to them. *	/	/	63	100				
	I generally enable my patients to ask all their questions.	3	4.7	61	95.3	1	33.7	2	66.7
	I always manage to give my patients the feeling that I will take enough time for their treatment.	/	/	62	100				
	I generally respond to what my patients say.	1	1.6	63	98.4	1	100	/	/
	I generally speak to my patients on an equal level.	1	1.6	63	98.4	1	100	/	/
	In all situations, I succeed in showing my patients that I take their concerns and wishes seriously.	/	/	63	100				
	I always explain the side effects and risks of treatments in detail without sugar-coating or leaving anything out.	2	3.2	61	96.8	1	0.5	1	0.5
	I always show my patients that I take their treatment suggestions/requests seriously.	/	/	62	100				
	I know my professional limits and communicate these to my patients whenever possible.	/	/	62	100				
	As a general rule, I do not judge my patients and their life decisions.	/	/	62	100				
	I always respond to my patients’ questions/treatment requests in a value-neutral manner and discuss them with an open mind.	1	1.6	61	98.4	/	/	1	0.5
Gender-specific communication	I always ask my patients for their pronouns. *	37	59.7	25	40.3	21	58.3	15	41.7
	On my/our website, you can already find an indication of the extent to which we use gendered language. *	39	66.1	20	33.9	20	55.6	16	44.4
	During the initial consultation, I always avoid gender-specific language, e.g., when I call patients to the examination room. *	43	69.4	19	30.6	25	64.1	14	35.9
	I always introduce myself with pronouns. *	50	79.4	13	20.6	17	38.6	27	61.4
	I regularly keep myself informed about current terminology and always talk about gender reassignment, for example. *	20	31.7	43	68.3	8	44.4	10	55.6
	I generally avoid phrases like “You can’t even tell”.	9	14.8	52	85.2	5	62.5	3	37.5
	I always give my patients the opportunity to indicate their gender individually at the start of treatment.	18	29.0	44	71.0	11	64.7	6	35.3
	I always manage to give my patients the feeling that they don’t have to explain their gender to me.	8	13.1	53	86.9	3	37.5	5	62.5

* Part of the original CommTrans Questionnaire [33]; Items without * were removed from the original CommTrans questionnaire due to ceiling effects.

**Table 3:** Doctors self-reported barriers towards trans-inclusive communication.

	Participants Who Said They Could Communicate Trans-Inclusively in the Future *	Participants Who Said They Could Not Communicate Trans-Inclusively in the Future *
Category (Number of Answers in This Category)	Example	Example
Necessity (n = 18)	Due to our location in the countryside, we have only treated two transgender patients so far, and I haven’t dealt with the topic enough so far.	Due to the rarity of transsexuality, it currently seems pointless to me to ask someone for their pronouns or to introduce mine. If a person would like to be addressed differently than what their name/appearance suggests, I will be happy to respond to this if they point it out and implement this according to their wishes.
Sample dependency (n = 3)	Many patients, especially older patients, do not understand the question about pronouns or consider this question to be completely unnecessary. In some example cases, even an explanation has not led to understanding from patients, who see such a question as irritating or even an affront.	Communication with the patient is subject to social norms corresponding to age/educational background/social background, which I, as the treating doctor, try to adapt to in the conversation. The topic of ‘gender’ is, therefore, not always part of the content of the conversation/I think about it in everyday life. Furthermore, some phrases may be appropriate for one group of patients but not for another. The question, for example, can, therefore, only be answered with insufficient precision.
Habit (n = 5)	Not a habit yet; I often don’t have to use pronouns in conversation.	That would just be difficult for me at the moment.
Structural barriers in practice (n = 4)	In practice, I would be very out of line with my colleagues. My boss has a rather conservative attitude towards this. I don’t have the courage to go through with it anyway.	I don’t have the ability to change the website.
Uncertainties in dealing with the topic (n = 5)	Although I take the topic seriously and want everyone to feel comfortable with their gender/identification with me, it hasn’t occurred to me so far to do this by asking for pronouns and avoiding gender-specific ones in speech, etc. I don’t know exactly how I could implement this respectfully/tactfully/empathically either.	I wouldn’t know how else to call patients without it being strange.
Limits of patient-centredness (n = 2)	In principle, there are wishes and suggestions that go so far outside the scope of the usual or scientific that they must be clearly formulated.	If I know what the better treatment is, I’m happy to explain why. Unfortunately, it’s not possible to be open-ended without letting people die.
Gender as a binary concept (n = 3)	/	Patients usually tell us their gender when they give their name. I differentiate between men and women as possible biological genders.
Transphobia (n = 2)	/	It is impossible to change your gender. Even mutilation does not make a man a woman. I can’t support someone with mental illness.

* Free text answers have been translated but not edited by the authors.

## Data Availability

The data used in this study are not available publicly due to data protection regulation.
